# Psychometric properties of the Portuguese Forms of Self-Criticizing/Attacking and Self-Reassuring Scale in older adults (FSCRS-OA)

**DOI:** 10.1192/j.eurpsy.2026.11577

**Published:** 2026-07-31

**Authors:** L. Lemos, D. Simões, H. Espírito-Santo, D. Andrade, D. Azedo, F. Nogueira, F. Daniel

**Affiliations:** 1 Instituto Superior Miguel Torga; 2 Centro de Inovação em Biomedicina e Biotecnologia, Universidade de Coimbra, Coimbra, Portugal

## Abstract

**Introduction:**

Population ageing heightens the relevance of self-relating processes for mental health. Self-criticism is linked to anxiety/depression, whereas self-reassurance may be protective. Evidence on the measurement of these constructs in Portuguese older adults remains limited.

**Objectives:**

To examine the psychometric properties of the Portuguese Portuguese Forms of Self-Criticizing/Attacking and Self-Reassuring Scale for use with older adults (FSCRS-OA), focusing on factor structure, internal consistency, and convergent evidence.

**Methods:**

We conducted a cross-sectional study with community-dwelling and social-care participants aged ≥65 years (*N* = 211; *M* = 80.9, *SD* = 7.6). Protocols included sociodemographics, FSCRS, Geriatric Anxiety Inventory, 8-item Geriatric Depression Scale, and WHOQOL-BREF. We tested latent structure and reliability and evaluated associations with anxiety, depression, quality of life and perceived health.

**Results:**

Analyses supported a correlated two-factor representation (self-criticism; self-reassurance) with satisfactory model fit. Internal consistency estimates were acceptable for both factors. Convergent patterns were theory-consistent: higher self-criticism aligned with greater anxiety/depressive symptoms, whereas self-reassurance related to fewer depressive symptoms and better quality of life/perceived health.

**Conclusions:**

Findings provide initial psychometric support for using the Portuguese FSCRS-OA in community-dwelling and social-care settings. Sample composition and the cross-sectional design are noted limitations. Broader and longitudinal samples are recommended to extend generalisability and temporal validity.

**Disclosure of Interest:**

None Declared

